# Presymptomatic Transmission of SARS-CoV-2 — Singapore, January 23–March 16, 2020

**DOI:** 10.15585/mmwr.mm6914e1

**Published:** 2020-04-10

**Authors:** Wycliffe E. Wei, Zongbin Li, Calvin J. Chiew, Sarah E. Yong, Matthias P. Toh, Vernon J. Lee

**Affiliations:** ^1^Ministry of Health, Singapore; ^2^National Centre for Infectious Diseases, Singapore; ^3^Saw Swee Hock School of Public Health, Singapore.

*On April 1, 2020, this report was posted online as an *MMWR *Early Release.*

Presymptomatic transmission of SARS-CoV-2, the virus that causes coronavirus disease 2019 (COVID-19), might pose challenges for disease control. The first case of COVID-19 in Singapore was detected on January 23, 2020, and by March 16, a total of 243 cases had been confirmed, including 157 locally acquired cases. Clinical and epidemiologic findings of all COVID-19 cases in Singapore through March 16 were reviewed to determine whether presymptomatic transmission might have occurred. Presymptomatic transmission was defined as the transmission of SARS-CoV-2 from an infected person (source patient) to a secondary patient before the source patient developed symptoms, as ascertained by exposure and symptom onset dates, with no evidence that the secondary patient had been exposed to anyone else with COVID-19. Seven COVID-19 epidemiologic clusters in which presymptomatic transmission likely occurred were identified, and 10 such cases within these clusters accounted for 6.4% of the 157 locally acquired cases. In the four clusters for which the date of exposure could be determined, presymptomatic transmission occurred 1–3 days before symptom onset in the presymptomatic source patient. To account for the possibility of presymptomatic transmission, officials developing contact tracing protocols should strongly consider including a period before symptom onset. Evidence of presymptomatic transmission of SARS-CoV-2 underscores the critical role social distancing, including avoidance of congregate settings, plays in controlling the COVID-19 pandemic.

Early detection and isolation of symptomatic COVID-19 patients and tracing of close contacts is an important disease containment strategy; however, the existence of presymptomatic or asymptomatic transmission would present difficult challenges to contact tracing. Such transmission modes have not been definitively documented for COVID-19, although cases of presymptomatic and asymptomatic transmissions have been reported in China (*1*,*2*) and possibly occurred in a nursing facility in King County, Washington (*3*). Examination of serial intervals (i.e., the number of days between symptom onsets in a primary case and a secondary case) in China suggested that 12.6% of transmission was presymptomatic (*2*). COVID-19 cases in Singapore were reviewed to determine whether presymptomatic transmission occurred among COVID-19 clusters.

The surveillance and case detection methods employed in Singapore have been described (*4*). Briefly, all medical practitioners were required by law to notify Singapore’s Ministry of Health of suspected and confirmed cases of COVID-19. The definition of a suspected case was based on the presence of respiratory symptoms and an exposure history. Suspected cases were tested, and a confirmed case was defined as a positive test for SARS-CoV-2, using laboratory-based polymerase chain reaction or serologic assays (*5*). All cases in this report were confirmed by polymerase chain reaction only. Asymptomatic persons were not routinely tested, but such testing was performed for persons in groups considered to be at especially high risk for infection, such as evacuees on flights from Wuhan, China (*6*), or families that experienced high attack rates.

Patients with confirmed COVID-19 were interviewed to obtain information about their clinical symptoms and activity history during the 2 weeks preceding symptom onset to ascertain possible sources of infection. Contact tracing examined the time from symptom onset until the time the patient was successfully isolated to identify contacts who had interactions with the patient. All contacts were monitored daily for their health status, and those who developed symptoms were tested as part of active case finding.

Clinical and epidemiologic data for all 243 reported COVID-19 cases in Singapore during January 23–March 16 were reviewed. Clinical histories were examined to identify symptoms before, during, and after the first positive SARS-CoV-2 test.

Records of cases that were epidemiologically linked (clusters) were reviewed to identify instances of likely presymptomatic transmission. Such clusters had clear contact between a source patient and a patient infected by the source (a secondary patient), had no other likely explanations for infection, and had the source patient’s date of symptom onset occurring after the date of exposure to the secondary patient who was subsequently infected. Symptoms considered in the review included respiratory, gastrointestinal (e.g., diarrhea), and constitutional symptoms. In addition, the source patient’s exposure had to be strongly attributed epidemiologically to transmission from another source. This reduced the likelihood that an unknown source was involved in the cases in the cluster.

## Seven Clusters of COVID-19 Cases Suggesting Presymptomatic Transmission

Investigation of COVID-19 cases in Singapore identified seven clusters (clusters A–G) in which presymptomatic transmission likely occurred. These clusters occurred during January 19–March 12, and involved from two to five patients each (Figure). Ten of the cases within these clusters were attributed to presymptomatic transmission and accounted for 6.4% of the 157 locally acquired cases reported as of March 16.

**FIGURE:**
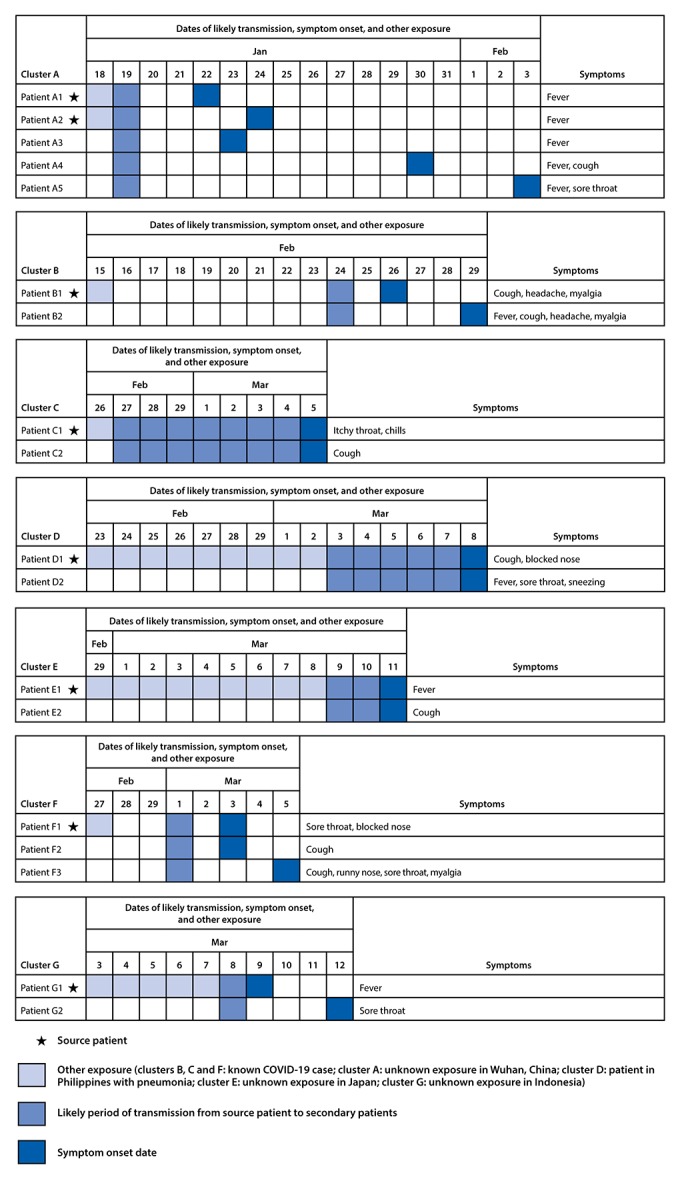
Seven COVID-19 clusters with evidence of likely presymptomatic SARS-CoV-2 transmission from source patients to secondary patients — Singapore, January 19–March 12, 2020

**Cluster A.** A woman aged 55 years (patient A1) and a man aged 56 years (patient A2) were tourists from Wuhan, China, who arrived in Singapore on January 19. They visited a local church the same day and had symptom onset on January 22 (patient A1) and January 24 (patient A2). Three other persons, a man aged 53 years (patient A3), a woman aged 39 years (patient A4), and a woman aged 52 years (patient A5) attended the same church that day and subsequently developed symptoms on January 23, January 30, and February 3, respectively. Patient A5 occupied the same seat in the church that patients A1 and A2 had occupied earlier that day (captured by closed-circuit camera) (*5*). Investigations of other attendees did not reveal any other symptomatic persons who attended the church that day.

**Cluster B.** A woman aged 54 years (patient B1) attended a dinner event on February 15 where she was exposed to a patient with confirmed COVID-19. On February 24, patient B1 and a woman aged 63 years (patient B2) attended the same singing class. Two days later (February 26), patient B1 developed symptoms; patient B2 developed symptoms on February 29.

**Cluster C.** A woman aged 53 years (patient C1) was exposed to a patient with confirmed COVID-19 on February 26 and likely passed the infection to her husband, aged 59 years (patient C2) during her presymptomatic period; both patients developed symptoms on March 5.

**Cluster D.** A man aged 37 years (patient D1) traveled to the Philippines during February 23–March 2, where he was in contact with a patient with pneumonia who later died. Patient D1 likely transmitted the infection to his wife (patient D2), aged 35 years, during his presymptomatic period. Both patients developed symptoms on March 8.

**Cluster E.** A man aged 32 years (patient E1) traveled to Japan during February 29–March 8, where he was likely infected, and subsequently transmitted the infection to his housemate, a woman aged 27 years (patient E2), before he developed symptoms. Both developed symptoms on March 11.

**Cluster F.** A woman aged 58 years (patient F1) attended a singing class on February 27, where she was exposed to a patient with confirmed COVID-19. She attended a church service on March 1, where she likely infected a woman aged 26 years (patient F2) and a man aged 29 years (patient F3), both of whom sat one row behind her. Patient F1 developed symptoms on March 3, and patients F2 and F3 developed symptoms on March 3 and March 5, respectively.

**Cluster G.** A man aged 63 years (patient G1) traveled to Indonesia during March 3–7. He met a woman aged 36 years (patient G2) on March 8 and likely transmitted SARS-CoV-2 to her; he developed symptoms on March 9, and patient G2 developed symptoms on March 12.

Investigation of these clusters did not identify other patients who could have transmitted COVID-19 to the persons infected. In four clusters (A, B, F, and G), presymptomatic transmission exposure occurred 1–3 days before the source patient developed symptoms. For the remaining three clusters (C, D, and E), the exact timing of transmission exposure could not be ascertained because the persons lived together, and exposure was continual.

## Discussion

This investigation identified seven clusters of COVID-19 in Singapore in which presymptomatic transmission likely occurred. Among the 243 cases of COVID-19 reported in Singapore as of March 16, 157 were locally acquired; 10 of the 157 (6.4%) locally acquired cases are included in these clusters and were attributed to presymptomatic transmission. These findings are supported by other studies that suggest that presymptomatic transmission of COVID-19 can occur (*1*–*3*). An examination of transmission events among cases in Chinese patients outside of Hubei province, China, suggested that 12.6% of transmissions could have occurred before symptom onset in the source patient (*3*).

Presymptomatic transmission might occur through generation of respiratory droplets or possibly through indirect transmission. Speech and other vocal activities such as singing have been shown to generate air particles, with the rate of emission corresponding to voice loudness (*7*). News outlets have reported that during a choir practice in Washington on March 10, presymptomatic transmission likely played a role in SARS-CoV-2 transmission to approximately 40 of 60 choir members.*

Environmental contamination with SARS-CoV-2 has been documented (*8*), and the possibility of indirect transmission through fomites by presymptomatic persons is also a concern. Objects might be contaminated directly by droplets or through contact with an infected person’s contaminated hands and transmitted through nonrigorous hygiene practices.

The possibility of presymptomatic transmission of SARS-CoV-2 increases the challenges of COVID-19 containment measures, which are predicated on early detection and isolation of symptomatic persons. The magnitude of this impact is dependent upon the extent and duration of transmissibility while a patient is presymptomatic, which, to date, have not been clearly established. In four clusters (A, B, F, and G), it was possible to determine that presymptomatic transmission exposure occurred 1–3 days before the source patient developed symptoms. Such transmission has also been observed in other respiratory viruses such as influenza. However, transmissibility by presymptomatic persons requires further study.

The findings in this report are subject to at least three limitations. First, although these cases were carefully investigated, the possibility exists that an unknown source might have initiated the clusters described. Given that there was not widespread community transmission of COVID-19 in Singapore during the period of evaluation and while strong surveillance systems were in place to detect cases, presymptomatic transmission was estimated to be more likely than the occurrence of unidentified sources. Further, contact tracing undertaken during this period was extensive and would likely have detected other symptomatic cases. Second, recall bias could affect the accuracy of symptom onset dates reported by cases, especially if symptoms were mild, resulting in uncertainty about the duration of the presymptomatic period. Finally, because of the nature of detection and surveillance activities that focus on testing symptomatic persons, underdetection of asymptomatic illness is expected. Recall bias and interviewer bias (i.e., the expectation that some symptoms were present, no matter how mild), could have contributed to this.

The evidence of presymptomatic transmission in Singapore, in combination with evidence from other studies (*9*,*10*) supports the likelihood that viral shedding can occur in the absence of symptoms and before symptom onset. This study identified seven clusters of cases in which presymptomatic transmission of COVID-19 likely occurred; 10 (6.4%) of such cases included in these clusters were among the 157 locally acquired cases reported in Singapore as of March 16. Containment measures should account for the possibility of presymptomatic transmission by including the period before symptom onset when conducting contact tracing. These findings also suggest that to control the pandemic it might not be enough for only persons with symptoms to limit their contact with others because persons without symptoms might transmit infection. Finally, these findings underscore the importance of social distancing in the public health response to the COVID-19 pandemic, including the avoidance of congregate settings.

**Summary**

**What is already known about this topic?**

Preliminary evidence indicates the occurrence of presymptomatic transmission of SARS-CoV-2, based on reports of individual cases in China.

**What is added by this report?**

Investigation of all 243 cases of COVID-19 reported in Singapore during January 23–March 16 identified seven clusters of cases in which presymptomatic transmission is the most likely explanation for the occurrence of secondary cases.

**What are the implications for public health practice?**

The possibility of presymptomatic transmission increases the challenges of containment measures. Public health officials conducting contact tracing should strongly consider including a period before symptom onset to account for the possibility of presymptomatic transmission. The potential for presymptomatic transmission underscores the importance of social distancing, including the avoidance of congregate settings, to reduce COVID-19 spread.

## Footnotes

* https://www.latimes.com/world-nation/story/2020-03-29/coronavirus-choir-outbreak.
